# The correlation between spiritual care competence and spiritual health among Iranian nurses

**DOI:** 10.1186/s12912-022-01056-0

**Published:** 2022-10-12

**Authors:** Akram Heidari, Zahra Afzoon, Morteza Heidari

**Affiliations:** 1 Spiritual Health Research Center, Qom University of Medical Sciences, Qom, Iran; 2 School of Allied Medical Sciences, Qom University of Medical Sciences, Qom, Iran; 3 School of Health and Religion, Qom University of Medical Sciences, Qom, Iran

**Keywords:** Spiritual health, Spiritual care competence, Nurses, Correlation analysis, Regression analysis, Iran

## Abstract

**Background:**

Considering the importance of spiritual aspects of human beings, spiritual care provision is increasingly recognized as a major duty of healthcare providers, particularly nursing staff. Spiritual care competence is necessary for the nurses to be able to provide spiritual care, but the competence itself is associated with other variables. This study aimed to investigate if the spiritual care competence of nurses is related to their spiritual health.

**Methods:**

A cross-sectional study was conducted with the participation of 172 practicing nurses in hospitals affiliated with Qom University of Medical Sciences, selected through stratified random sampling. Participants completed the Persian versions of the Spiritual Health Questionnaire (Amiri) and the Spiritual Care Competence Scale (Van Leeuwen). To examine the correlation between nurses’ spiritual health and spiritual care competence, a Spearman coefficient was used and a linear regression analysis was done to determine the predictability of the spiritual care competence of the nurses. The data were analyzed using SPSS v.23 and the significance level was set at 0.05.

**Results:**

The participants showed a mean (SD) score of 108.93 (19.04) on spiritual care competence and 213.38 (16.49) on spiritual health. Spiritual care competence of nurses showed no significant relationship with demographic characteristics and their spiritual health had a significant relationship with gender only. Correlation analysis revealed a significant relationship between spiritual health and spiritual care competence and their subscales. Moreover, the linear regression analysis indicated that the nurses’ performance regarding spiritual health can predict their spiritual care competence.

**Conclusion:**

The study revealed that the spiritual care competence of nurses is correlated with their spiritual health and performance as a subscale of spiritual health can predict their spiritual care competence. Thus, it can be concluded that the spiritual health of nurses is an important factor in providing spiritual care for patients and meeting their spiritual needs.

## Background

Spirituality has been favored in recent decades as a component of holistic care [1]. It is believed that the healthcare providers’ responsibility to patients exceeds their physical health and encompasses other aspects including the spiritual dimension of health. This is more evident in nursing in which the inclusion of spiritual issues could bring an added value through a compassionate and more humane approach to nursing care [2]. Despite the biomedical paradigm in which non-physical matters such as spirituality were not considered relevant, the holistic view integrates spirituality as a component of medicine and nursing that could lead to better health outcomes. Holistic view provides an explanation for one’s relationships with self, others, nature, and Spirit (God) [3] and results in a better state of peace and comfort for patients. From a holistic perspective, the beliefs, values, and attitudes of patients are regarded as potential resources contributing to the health of individuals if they are utilized effectively. In other words, the spiritual characteristics of any individual can be used as a strength and resource for better health, and without adequate attention to the spiritual needs of patients, a comprehensive approach to human health is impossible [4]. Spiritual care is an important part of holistic care that could facilitate the use of spiritual resources for the sake of the patient’s well-being. To provide spiritual care, nurses should recognize, respect, and meet patients’ spiritual needs [5]; facilitate their participation in religious rituals; communicate through listening and talking with clients; and accompany them by caring, supporting, and showing empathy [6]. As a result, patients experience a better sense of well-being through finding meaning and purpose in their illness and overall life [7]. They may also need to refer patients with severe spiritual needs to chaplains as professional spiritual care providers [8].

Various consequences of spiritual care including its effects on better quality of life [9], anxiety reduction [10], and spiritual health improvement [11], especially in palliative care [12] and for patients with cancer [13] and other end-stage patients have been evidenced. The advent of the Coronavirus disease 2019 (COVID-19) pandemic in recent years underscored the importance of spiritual care once more [14].

Spirituality plays a more important role in faith-based communities including Iran and the need to address spirituality is of utmost importance. Therefore, there is sufficient evidence on the benefits of spiritual care to convince us of the necessity of spiritual care competence for healthcare providers, particularly nurses [15].

On the other hand, there is a growing tendency toward spirituality among patients who expect healthcare providers to address their spiritual needs [16]. The patients’ needs and expectations are acknowledged by a lot of healthcare providers, but spiritual care is not commonly addressed as an integral part of healthcare. The main cause of this failure is the missing or inadequate abilities of the healthcare providers including nurses to provide spiritual care. In other words, the spiritual care competence of nurses as a prerequisite to providing spiritual care is necessary to be attended to and developed [17, 18] for which, a variety of training programs have been designed and implemented [19].

Spiritual care competence is an active and ongoing process, consisting of increasing awareness of the patient’s values, empathetic understanding of their worldviews, and capability for individualized interventions for each patient [20]. It includes assessment, planning, intervention, and evaluation of spiritual care in addition to intrapersonal and interpersonal spirituality [21]. Spiritual care competence consists of a set of knowledge, skills, and attitudes that enable nurses to practice holistic care in their caring behavior, while nurses’ caring behavior is closely related to their spiritual health [22].

Spiritual health is associated with the meaning and purpose of life, transcendence, faithfulness, and interconnectedness, and constitutes an essential dimension of holistic being [23]. Nurses’ spiritual health has a positive relationship with their attitude toward spiritual care [24], which in turn, could lead to an improved level of performance in spiritual care provision. Spiritual health has a close relationship with spiritual care. Spiritual care is provided to improve the spiritual health of patients and at the same time, the spiritual health of the healthcare providers is an empowering factor in providing spiritual care. So, the personal spirituality and spiritual health of nurses can be assumed to be closely related to spiritual care competence. Spiritual health not only enables nurses to provide better spiritual care, but leads to personal and professional consequences such as happiness, resilience, higher quality of life, better work performance, and less burnout [25]. The theoretical framework of this study is based on the iceberg mode of competence [26]. According to this model, competence consists of not only knowledge and skills as the visible components, but also motives, traits and self-image that are invisible yet important components of competence. In fact, motives and traits are the internal drives and individual characteristics that lead to one’s behaviors in different situations and self-image is a sense of identity that is rooted in one’s values. These deeper layers of competence are closely related with one’s spirituality and spiritual health. They provide intrinsic motivation that leads to professional performance. Given the importance of spiritual care competence and the role of one’s inner spirituality in this regard, this study hypothesized that nurses’ spiritual health is positively related to their spiritual care competence. Therefore, the present study aimed to analyze the correlation between the spiritual health and spiritual care competence of Iranian nurses.

## Methods

### Design and setting

A cross-sectional study was carried out in six hospitals affiliated with Qom University of Medical Sciences in Qom, a central city in Iran, in 2021. All hospitals were academic centers varying from general to specialized.

### Sampling

The study population consisted of nurses working in any of the six academic hospitals affiliated with Qom University of Medical Sciences. The participants were selected through stratified random sampling. To determine the required sample size with the assumption of α (two-tailed) = 0.01, β = 0.10, and r = .29 [27], the sample size of 170 nurses was estimated using the related formula. Taking into account the participants’ attrition rate of 10%, 187 nurses were selected for the study. The calculated sample size was first distributed according to the number of practicing nurses in each of the six hospitals and then according to male and female nurses in each hospital. Finally, simple random sampling was performed. The inclusion criteria consisted of a minimum degree of B.S. in nursing and a full-time occupation at hospital. So, in cases of receiving no response despite follow-up messages, the samples were substituted with nurses with similar characteristics who met the inclusion criteria.

### Instrument overview

For the data gathering procedure, we used a web-based questionnaire consisting of three parts: (1) the demographic characteristics including gender, age, education level, marital status, the workplace hospital, working experience years, ward, and position (nurse, head nurse, supervisor). (2) the spiritual health questionnaire for the Iranian population [27]. (3) the spiritual care competence scale [28].

### Spiritual health questionnaire for the Iranian population

This 48-item questionnaire was designed by Amiri and her colleagues in 2015 and its validity and reliability were confirmed. The questionnaire consists of two subscales. The first 28 questions address insight and tendency that are scored on a 5-option Likert scale: *strongly disagree = 1*, *disagree = 2*, *undecided = 3*, *agree = 4*, and *strongly agree = 5*. The 20 questions that follow assess the performance of the respondents according to their behavior within the last year on a 5-option Likert scale: *never = 1*, *rarely = 2*, *sometimes = 3*, *usually = 4*, *always = 5*. Cronbach’s alpha coefficients calculated exceeded the minimum reliability standard of 0.70 and confirmed the internal consistency of scales and subscales [27]. The Cronbach’s alpha of the scale was calculated for this study as 0.94.

### Spiritual Care Competence Scale

The questionnaire was developed by Van Leeuwen and his colleagues in 2009. It consists of 27 statements in six subscales: (1) assessment and implementation of spiritual care, (2) professionalization and improving the quality of spiritual care, (3) personal support and patient counseling, (4) referral to professionals, (5) attitude towards the patient’s spirituality and (6) communication. Each subscale consisted of 2 to 6 questions that were scored on a 5-point Likert scale: *strongly disagree = 1*, *disagree = 2*, *undecided = 3*, *agree = 4*, and *strongly agree = 5* [28]. The validity and reliability of the Persian version of the scale were determined by Khalaj and his colleagues with Cronbach’s alpha of 0.77 for the whole scale and between 0.65 and 0.85 for different subscales [29]. The Cronbach’s alpha of the scale was calculated as 0.96 for this study.

### Data analysis

After being collected, the data were analyzed in SPSS v.23 using descriptive statistics (frequency, percentage, mean, and standard deviation). To investigate the relationship between the participants’ spiritual care competence and their spiritual health and demographic variables, the researchers used the t-test, Mann-Whitney U test, Kruskal Wallis test, Spearman’s correlation coefficient, and ANOVA. Before performing the tests, the normality of the data distribution was checked using the One-Sample Kolmogorov-Smirnov test. It was shown that the distribution of the variables of age and spiritual care competence did not have a significant difference with normal distribution (p > .05), while spiritual health and work experience showed a significant difference with normal distribution (p < .05).

After performing correlation analysis through the Spearman coefficient, the variables that correlated with spiritual care competence (p < .20) entered into a multiple linear regression model using enter method. The data were analyzed with spiritual care competence as the dependent variable and the two subscales of spiritual health (insight and tendency and performance) as independent variables. Before using linear regression, the researchers tested the data to assure that the following assumptions are met: normality of the data, independence of residuals, residual normality, and multicollinearity. The level of significance was set at P < .05.

## Results

A total of 180 nurses participated in the study. Eight questionnaires were excluded because of multiple missing values and thus 172 questionnaires entered the analysis process. The participants’ age ranged from 22 to 60 years and their work experience from 1 to 30 years. The participants worked in six hospitals affiliated with Qom University of Medical Sciences. The distribution of the participants was proportionate to the size and the staff number of each hospital, ranging from 12 to 50. All the participants were Shia Muslims, and thus there was no diversity among the participants in terms of religion. The demographic characteristics of the participants are summarized in Table 1.

**Table 1:** Demographic characteristics of the participants

Variable	Mean (SD)	Frequency (%)
**Age**	34.86 (8.16)	
**Work experience**	9.65 (7.13)	
**Gender**		
Female		113 (66.5)
Male		57 (33.5)
**Marital status**		
Married		118 (69.8)
Single		51 (30.2)
**Education level**		
Bachelor		146 (86.9)
Master		20 (11.9)
Ph.D		2 (1.2)
**Position**		
Nurse		145 (86.8)
Head-nurse		9 (5.4)
Clinical supervisor		8 (4.8)
Training supervisor		5 (3)
**Ward**		
Internal		40 (29.7)
Emergency		28 (20.7)
Intensive care		28 (20.7)
Surgery		24 (17.8)
Nursing office		15 (11.1)

The participants showed a mean (SD) spiritual health of 213.42 (19.04) (the maximum score possible was 240) and the mean (SD) spiritual care competence of 109.08 (16.49) (the maximum score possible was 135). Table 2 summarizes the spiritual health and spiritual care competence scores of the participants in terms of different demographic characteristics. The results indicated a significant relationship between spiritual health and gender, while there was no significant relationship between spiritual care competence and demographic variables including gender, marital status, education level, working ward, and position.

**Table 2:** Scores of spiritual health and spiritual care competence in relation to demographic characteristics

Variable	Values	N	Spiritual health				Spiritual care competence						
			**Mean (SD)**		**P**			**Mean (SD)**			**P**		
**Gender**	Female	113	216.87 (16.18)		0.003^*^			111.10 (14.71)			0.520^**^		
	Male	57	206.44 (22.53)					104.63 (19.07)					
	Sum	170											
**Marital Status**	Single	51	209.06 (23.21)		0.190^*^			104.35 (17.75)			0.660^**^		
	Married	118	215.05 (16.85)					110.68 (15.58)					
	Sum	169											
**Education Level**	Bachelor	146	213.00 (19.40)		0.256^***^			108.08 (17.02)			0.322^****^		
	Master	20	217.77 (17.91)					113.09 (12.35)					
	Ph.D	2	205.89 (10.15)					118.23 (8.81)					
	Sum	168											
**Ward**	Surgery	24	209.28 (16.74)		0.160^***^			101.75 (17.94)			0.230^****^		
	Internal	40	215.91 (17.14)					111.32 (13.95)					
	Emergency	28	204.63 (26.11)					102.44 (17.24)					
	Intensive care	28	214.76 (19.38)					108.55 (14.18)					
	Nursing office	15	218.28 (19.67)					114.40 (14.48)					
	Sum	135											
**Position**	Nurse	145	212.69 (19.04)		0.372			108.17 (16.09)			0.378^****^		
	Head Nurse	9	222.08 (12.85)					115.77 (11.51)					
	Clinical Supervisor	8	218.91 (20.55)					114.87 (13.77)					
	Training Supervisor	5	207.80 (28.17)					105.33 (34.93)					
	Sum	167											

*. Mann Whitney U test

**. T- test

***. Kruskal Wallis test

****. ANOVA test

To explore the interrelationship between spiritual health and spiritual care competence, the correlation analysis was carried out through Spearman’s rho test. The results showed a significant relationship between spiritual health and spiritual care competence. In this way, the findings of the study lead to the approval of the research hypothesis. Moreover, the subscales of the two variables were studied regarding their correlation. The correlation between spiritual health and spiritual care competence and their subscales is indicated in the correlation matrix (Table 3).

**Table 3:** Correlation matrix of spiritual care competence and spiritual health

Variables	1	2	3	4	5	6	7	8	9	10
1- assessment and implementation of spiritual care	1.000									
2- professionalization and improving the quality of spiritual; care	0.584^*^	1.000								
3- personal support and patient counseling	0.449^*^	0.740^*^	1.000							
4- referral to professionals	0.388^*^	0.629^*^	0.779^*^	1.000						
5- attitude toward the patient’s spirituality	0.349^*^	0.623^*^	0.715^*^	0.696^*^	1.000					
6- communication	0.296^*^	0.604^*^	0.718^*^	0.636^*^	0.802^*^	1.000				
**7- Spiritual Care Competence**	0.626^*^	0.878^*^	0.902^*^	0.825^*^	0.832^*^	0.797^*^	1.000			
8- Insight and tendency	0.343^*^	0.361^*^	0.359^*^	0.356^*^	0.321^*^	0.360^*^	0.421^*^	1.000		
9- Performance	0.510^*^	0.542^*^	0.536^*^	0.479^*^	0.437^*^	0.409^*^	0.593^*^	0.620^*^	1.000	
**10- Spiritual health**	0.475^*^	0.491^*^	0.479^*^	0.448^*^	0.401^*^	0.415^*^	0.548^*^	0.898^*^	0.886^*^	1.000

*. Correlation is significant at the 0.001 level (2-tailed)

**Fig. 1:**
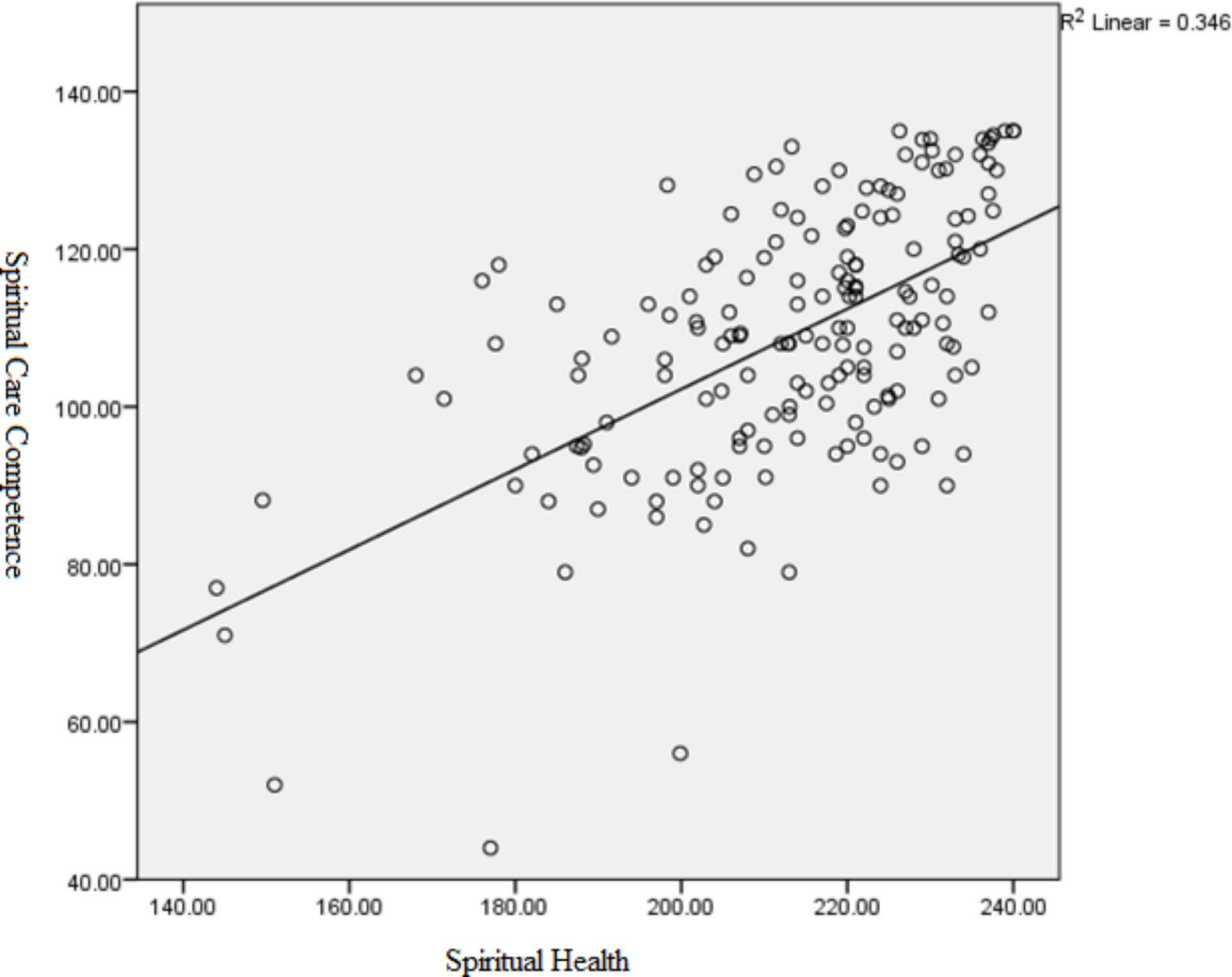
Correlation between spiritual health and spiritual care competence

Figure 1 illustrates the above-mentioned correlation in a scatter plot. As shown, the values of the vertical axis (spiritual care competence) increase with the increase of the values of the horizontal axis (spiritual health), and thus, higher spiritual care competence is associated with higher spiritual health. Moreover, the position of the data points shows that the spiritual health and spiritual care competence of the respondents tend to occur mostly at the above-average values.

To explain the spiritual care competence of the nurses, a linear regression analysis was performed. The correlation of the subscales “insight and tendency” and “performance” with the spiritual care competence (p < .05, r = .42) and (p < .05, r = .59) respectively suggested a significant positive correlation. Nevertheless, the regression analysis revealed that the nurses’ insight and tendency toward spiritual health could not act as a predictor of their spiritual care competence, while their spiritual health performance significantly explained 38% of “spiritual care competence” (Table 4).

**Table 4:** Results of linear regression analysis of predictors of spiritual care competence

Predictor		Unstandardized Coefficients		Standardized Coefficients	T	Sig.	95.0% Confidence Interval for B	
		B	Std. Error	Beta			Lower Bound	Upper Bound
	Spiritual care competence (Constant)	10.011	11.522		0.869	0.386		
	Insight and tendency	0.174	0.111	0.119	1.567	0.119	− 0.045	0.394
	Performance	0.899	0.126	0.543	7.155	0.000	0.651	1.147

R2 = 0.388, Adjusted R2 = 0.381

## Discussion

This study assessed the correlation between the spiritual care competence of nurses with their spiritual health. The findings revealed that gender had a significant relationship with the spiritual health of the nurses, while spiritual care competence showed no significant relationship with demographic characteristics. There was a significant positive relationship between spiritual health and spiritual care competence and most of their subscales. Moreover, it was shown that performance as a subscale of spiritual health could predict the spiritual care competence of the nurses.

In previous studies, Adib-Hajbaghery found no significant relationship between spiritual care competence of nurses and their gender, marital status, position, and level of education [30]. Some studies implied that the overall score of nurses in terms of spiritual care competence was significantly related to the working experience of the nurses and their position so that nurses had a higher score than the head nurses [31]. Other studies indicate a reverse correlation between nurses’ years of service and their spirituality [32]. This may be partly due to the recent trends in nursing education through which the younger nurses are more acquainted with spiritual issues of nursing.

The correlation between spiritual health and spiritual care competence [33] and a positive relationship of spiritual care competence with spiritual well-being and spiritual care perceptions or perspective toward spiritual care has been shown in previous studies too [34–36]. No other study was found to asses the correlation between the subscales of the spiritual care competence and spiritual health. This can be regarded a novel finding that suggested that while both performance and insight and tendency as the subscales of spiritual health have a significant relationship with spiritual care competence, performance is a more important determinant factor. This importance lies in the fact that spiritual performance originates from a deeper personal spirituality compared with knowledge and tendency. This is consistent with another study showing a positive significant correlation between the spirituality of nurses with their understanding and practice of spiritual care [37]. The relationship between spiritual health and spiritual care competence has important implications. Spiritual care considered a commitment of a nurse, is likely to be attended by nurses, but in different ways and levels depending on whether it is regarded as a professional commitment or is rooted in the inner motivation of oneself. In other words, spirituality is an inner motive that facilitates nurses’ practice in spiritual care [32].

Another study indicated the relationship between nurses’ spiritual health and their attitudes toward spiritual care, as well as professional commitment and caring. Nurses’ positive attitudes toward spiritual care were shown as a mediator of the spiritual health professional commitment and spiritual health caring relationships. Findings indicated that nurses’ spiritual health should be regarded as an important value and belief system that can affect their professional performance [38].

To fill the gap between the existing and desired spiritual care competence of nurses, training programs have been developed and implemented that endorse the consequences of this training on spiritual health too [39]. A systematic review study of the spiritual care training programs indicated the outcomes of spiritual care in terms of spiritual assessment, spiritual interventions, and intrapersonal and interpersonal spirituality [40]. Enhancing Nurses’ and Midwives’ Competence in Providing Spiritual Care through Innovative Education and Compassionate Care is a project network that offers a unique resource for nursing and midwifery educators and students and helps them develop a new awareness and enhance their expertise in the area of spiritual care [41]. Successful spiritual care education is conditional on different considerations from student selection criteria to the teaching and learning environment, especially the clinical environment, student assessment, and attention to the individual differences of students [42].

The association between spiritual health and spiritual care competence lies in the fact that competence encompasses a variety of knowledge, attitude, skills, and behaviors. The inner spirituality of care providers is an influencing factor determining the level of spiritual care competence. Therefore, in general, those who have higher levels of spiritual health demonstrate higher levels of perceived spiritual care competence. This denotes that the cultivation and strengthening of one’s spiritual life relate to the provision of more holistic nursing care that includes attending to the spiritual needs of patients.

## Limitations

The main limitation of this study was the reluctance of the nurses to participate in the study. It seems that the COVID-19 crisis intensified the situation as a result of the work overload and other consequences on nurses. We tried to motivate and encourage the nurses through face-to-face persuasion. When needed, we substituted the participants with nurses with equivalent characteristics.

## Conclusion

This study aimed to assess the relationship between the spiritual care competence of the nursing staff with their spiritual health. Spiritual care competence is a prerequisite for providing spiritual care and it is needed to be addressed and improved if nurses are expected to provide spiritual care. The finding of the present study regarding the close relationship between the spiritual care competence with spiritual health of nurse particularly their performance in this regard highlights the necessity of addressing and improving the spiritual health of the nurses. In other words, not only the interventions to increase the spiritual care competence among nurses is necessary as a means of improving the holistic approach to healthcare, but any effort to improve the spiritual health of the staff should be welcome.

## Data Availability

The data are available and deliverable from the corresponding author on reasonable request.
